# Functional Invertebrate Prey Groups Reflect Dietary Responses to Phenology and Farming Activity and Pest Control Services in Three Sympatric Species of Aerially Foraging Insectivorous Birds

**DOI:** 10.1371/journal.pone.0114906

**Published:** 2014-12-15

**Authors:** Grzegorz Orłowski, Jerzy Karg, Grzegorz Karg

**Affiliations:** 1 Institute of Agricultural and Forest Environment, Polish Academy of Sciences, Poznań, Poland; 2 Department of Nature Conservation, Faculty of Biological Sciences, University of Zielona Góra, Zielona Góra, Poland; Point Blue Conservation Science, United States of America

## Abstract

Farming activity severely impacts the invertebrate food resources of farmland birds, with direct mortality to populations of above-ground arthropods thorough mechanical damage during crop harvests. In this study we assessed the effects of phenological periods, including the timing of harvest, on the composition and biomass of prey consumed by three species of aerial insectivorous birds. Common Swifts *Apus apus*, Barn Swallows *Hirundo rustica* and House Martins *Delichon urbica* breed sympatrically and most of their diet is obtained from agricultural sources of invertebrate prey, especially from oil-seed rape crops. We categorized invertebrate prey into six functional groups, including oil-seed rape pests; pests of other arable crops; other crop-provisioned taxa; coprophilous taxa; and taxa living in non-crop and mixed crop/non-crop habitats. Seasonality impacted functional groups differently, but the general direction of change (increase/decrease) of all groups was consistent as indexed by prey composition of the three aerial insectivores studied here. After the oil-seed rape crop harvest (mid July), all three species exhibited a dietary shift from oil-seed rape insect pests to other aerial invertebrate prey groups. However, Common Switfts also consumed a relative large quantity of oil-seed rape insect pests in the late summer (August), suggesting that they could reduce pest insect emigration beyond the host plant/crop. Since these aerially foraging insectivorous birds operate in specific conditions and feed on specific pest resources unavailable to foliage/ground foraging avian predators, our results suggest that in some crops like oil-seed rape cultivations, the potential integration of the insectivory of aerial foraging birds into pest management schemes might provide economic benefits. We advise further research into the origin of airborne insects and the role of aerial insectivores as agents of the biological control of crop insect pests, especially the determination of depredation rates and the cascading effects of insectivory on crop damage and yield.

## Introduction

Aerially foraging vertebrate insectivores, including birds and bats, are heavily dependent on the abundance and availability of flying insects, and display substantial plasticity towards prey type and feeding micro-habitats [1]–[10]. Overall, some species or taxa of invertebrates (including the prey of aerial vertebrate insectivores) show a strong preference for certain land uses, habitats or even individual crop types occurring in broadly agricultural landscapes, resulting in habitat-specific densities [11]–[17]. The community of non-target wild animals occurring within arable landscapes in Europe is undergoing dramatic changes as a result of agricultural practices. These species can be especially vulnerable during crop harvest, when they may suffer direct mortality or be forced to move to adjacent non-crop habitats [17], [18]. Since invertebrates are of primary importance as food resources for farmland birds, including some species of declining aerial insectivores, assessment of the effects of agricultural activity on predators’ diet composition is important [19]–[21]. This research also has implications for understanding predator-prey trophic interactions in communities of organisms living in intensively managed farming landscapes [22], [23]. This research also has implications for understanding the dynamic of the processes within food webs and prey-predator trophic interactions in communities of organisms living in intensively managed farming landscapes [22], [23].

Many empirical studies have documented seasonal and crop-specific changes in soil cultivation regimes, fertilization and pesticide applications with respect to the number and biomass of arthropod resources of farmland birds, reviewed in [19], [20], [24]. However, there are few studies on the direct dietary responses of insectivorous birds (as a whole group) to external environmental conditions, including intensive agricultural practices, cf. [21]. There are a relatively large number of studies examining relationships between aerial invertebrate abundance and agricultural land-uses and weather conditions [25]–[28], particularly in the context of the food density of some declining aerial insectivorous feeding birds, such as Barn Swallows *Hirundo rustica* [6], [7]. To the best of our knowledge, no work has been done to date to find out directly how the diet of the sympatric community of three aerial insectivores breeding in rural parts of Europe (Common Swift *Apus apus*, Barn Swallow and House Martin *Delichon urbica*) changes in the course of the breeding season and under the severe environmental impact of the crop harvest. These three species occur widely in the northern part of the western Palaearctic and currently breed almost exclusively within human settlements and urbanised areas [29], [30]. Relatively extensive knowledge exists regarding the food composition of these three species based on the classical taxonomic classification of invertebrate prey, as compiled in [29], [30]. Under certain circumstances, in rural areas or during migration, for example, all three can utilise similar prey groups and their diets overlap, especially during bad weather [1], [29], [31].

In this study we assessed the effects of phenological periods, including the timing of the harvest of the main crops, on the composition and biomass of invertebrate prey in the diet of nestlings of three species of aerially foraging insectivorous birds – Common Swifts, Barn Swallows and House Martins. These three species make up a specific community of aerial predators varying in the abundance of individual species. They breed sympatrically and use agriculturally subsidised resources of invertebrate prey, especially from extensive oil-seed rape cultivation. The key element of our analysis of the aerial invertebrate prey community is the functional grouping of individual taxa/species/genera according to the ecological features of individual invertebrate species, as some researchers have recently proposed, see: [32], [33]. Thus, taking previous concepts of functional biodiversity within agricultural landscapes into consideration, cf. [34], [35], we classified all identified arthropod prey into six consistent functional groups of invertebrates with respect to their habitat, food, or association with crop habitat, cf. [17], (see below). Such a classification of invertebrate prey allows us to draw a relatively uniform picture of changes in the community of invertebrates living in various habitats of an agricultural landscape under different environmental stressors. It might provide some valuable estimates for the analysis of ecosystem services provided by aerial insectivores [33]. Our previous dietary study conducted on these three aerial insectivores showed that during whole breeding seasons the most numerous prey types of each bird species were pest insects of oil-seed rape crops [31]. Hence, as aerial insectivores are potentially important in the biological control of insect pests [36], [37], [33], we focus in this study on seasonal changes in the proportions of these invertebrates, dividing them into two separate groups according to their host crops: oil-seed rape pests and pests of other arable crops. The investigation presented in this paper (with the practical classification and seasonal changes of invertebrate prey groups) complements both our previous dietary study on the community of these three aerial insectivores [31], as well as earlier comparative studies on dietary overlap among some European aerially foraging insectivorous birds [1]–[5], [38], (cf. [42] for comments on specific methodological differences between all these investigations). We discuss our findings in the context of previously published studies on resources and diets of farmland insectivorous birds and draw practical conservation conclusions on the feeding ecology and biological control of insect pests provided by aerial insectivores in dynamic agricultural systems. This partly addresses the need highlighted in recent studies to quantify the economic importance of vertebrate insectivory [33], [37].

## Material and Methods

**Figure 1:**
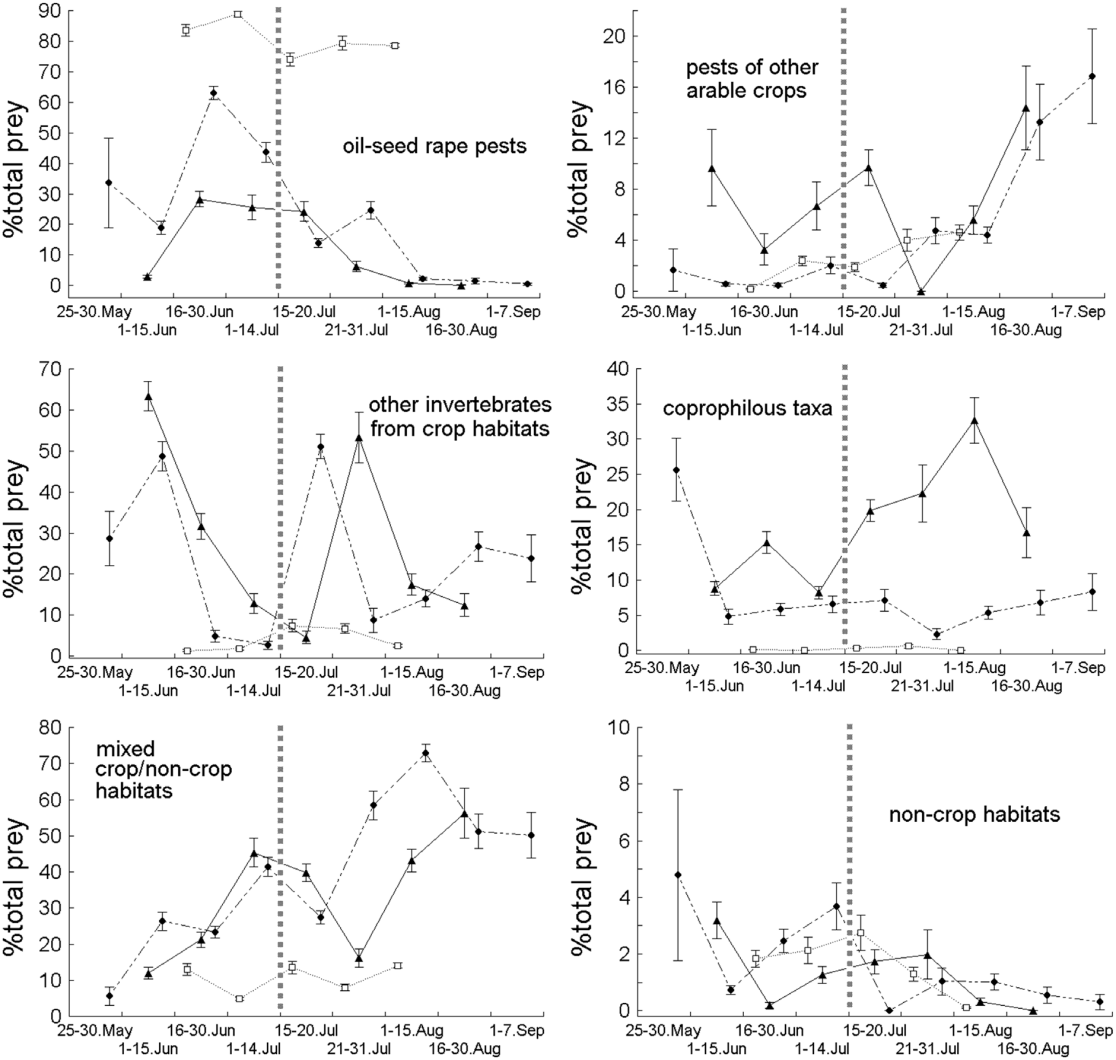
Percentage composition (average±SE) of the number of six functional aerial invertebrate prey groups identified in individual faecal sacs of nestlings of Common Swifts *Apus apus* (□), Barn Swallows *Hirundo rustica* (▴) and House Martins *Delichon urbica* (•) in various periods of the breeding season 2012; note different scales on y-axes; the harvest of oil-seed rape crops (15–20 July) is indicated by the vertical dotted grey line; for Common Swifts, Barn Swallows and House Martins the number of faecal sacs analysed in consecutive periods was: 25–30 May (0/0/4), 1–15 June (0/27/62), 16–30 June (10/76/33), 1–14 July (17/7/35), 15–20 July (66/35/27), 21–31 July (9/14/13), 1–15 August (10/65/45), 16–31 August (0/22/18), 1–7 September (0/0/35).

We investigated the diets of nestling Common Swifts, House Martins and Barn Swallows in 2012 by identifying prey items from faecal sacs collected under active nests of these species. All three species breed in Stary Gołębin, a village in south-western Poland located in an agricultural region of western Wielkopolska (see the geospatial data in the S1 File). Overall, within the breeding community of the three aerial insectivores in the entire village, House Martins were the most numerous (*c.* 95 pairs; 76% in the whole community), Barn Swallows less numerous (up to *c.* 20 pairs; 16%), and Common Swifts the least numerous (up to *c.* 10 pairs; 8%). For this detailed dietary study we chose the nests of the three species distributed within a small, 3 ha plot; the maximum distance between nests was approximately 160 m (see S1 File). In 2011 a dietary study of these three sympatric aerial insectivores was conducted in the same area; more details on the nesting sites of the three species are given elsewhere [31]. Prior to the collection of faecal sacs we sought and obtained permission from the owners of the buildings where the nests of the examined birds were located. We collected faecal sacs accumulated over 1–10-day intervals under occupied nests of the three species: from three Common Swift nests located in the ventilation holes of a low building, two large breeding colonies of House Martins with 60 and 35 nests respectively located on two four-storey blocks of flats, and four Barn Swallow nests situated in four small farm buildings, two of which housed pigs or piglets. In addition, since only the faeces of House Martins and Barn Swallows formed visible piles we sampled one-day faecal samples (i.e. faecal sacs sampled during one day from morning to evening) from these two species by placing sheets of paper on the ground under their nests. In practice we placed one sheet under Barn Swallow nests and 2–3 sheets under the nests of House Martins. Owing to the large number of House Martin nests, their faecal sacs were always sampled in various parts of the colony, so very likely the faeces accumulated on one sheet of paper came from several different broods. Over the whole time span we sampled faeces uniformly from all nests of Common Swifts and Barn Swallows. Faecal sacs of Common Swifts were collected between 28 June and 7 August (their faecal sacs were sampled from the ground since they were scattered over a larger area), of House Martins between 25 May and 7 September, and of Barn Swallows between 31 May and 21 August. Overall, during the entire breeding season we collected 112 faecal sacs from Common Swifts, 272 from House Martins and 246 from Barn Swallows. Faecal sample size numbers per time interval are provided in text of Fig. 1.

On the basis of published findings we assumed that a radius of 500 m around the centre of the sympatric breeding site of these insectivores would correspond to the distance flown in search of food by adult Barn Swallows and House Martins when rearing their young [2], [39]–[41]. In calm weather, Common Swifts usually feed close to the colony [36; our observations], but during bad weather, their foraging range may extend to several kilometres [cf. 30]. In addition, our previous findings (from 2011) showed that the staple diet of Common Swifts were oil-seed rape insect pests (76% of all consumed prey), which most likely were taken over rape crops growing near the study area [31]. Hence we assumed that the management of the crops within a 500 m radius could directly affect the availability of some prey groups (especially invertebrate taxa living in a crop habitat), and finally their contribution to the diets of the three studied species. The village has a large farm with cattle (1 370 head), pigs (25 head) and ca 50 poultry. The main land-use within a radius of 500 m around the centre of the sympatric breeding sites (see S1 File) was crops (67.6% of the total area), including maize *Zea mays* (three fields; 33.2%), winter oil-seed rape *Brassica napus* L. (one field; 18.1%), alfalfa (one field; 4.3%) and spring triticale (one field; 0.6%); other arable land consisted of several very small fields with various vegetable crops (11.4%); a wooded area; one relatively large manorial park with a deciduous tree stand (15.9%); a built-up area, including farm buildings (12.9%); permanent non-crop vegetation, including lines of trees, hedgerows and perennial (ruderal) vegetation (3.6%).

All crops within a 500 m radius were managed (over several years) using conventional amounts of agrochemicals, including pesticides and fertilisers. The land holder (Top Farms Wielkopolska Co., Poland) supplied management data on agricultural practices on individual fields for the study years (2011/2012). One winter oil-seed rape (cultivar: PRW 31 F-1) field was sown between 20–25 September 2011; in spring 2012 there were 2 herbicide, 6 fungicide and 5 insecticide applications between March and May (Ammo Supre, Pyrinex 480 Ec, Proteus 110 Od) targeting stem weevils *Ceutorhynchus* spp. and pollen beetles *Meligethes* spp. The crop was harvested between 15 and 20 July. The maize crops were treated with herbicide 3–4 times, with fungicide 0–3 times and with insecticide 0–1 time; they were harvested in October. The winter triticale field was treated twice with herbicide, 3 times with fungicide and once with insecticide; the crop was harvested between 10 and 20 August. No pesticides were applied to the alfalfa field.

### Determination of diet, classification of invertebrate prey and data analysis

The method of faecal analysis used to determine the taxonomic composition of the diet of the studied birds is presented elsewhere in detail [31], [42]. The differences in the taxonomic composition of the diets of these species breeding in this same area in 2011 were analysed previously [31]; those faecal samples were not re-used or pooled with the data from 2012, however. To determine the number of prey items belonging to a particular species, we applied the rule of summation of different chitin parts to the level of one individual, in accordance with previous studies [31], [42], [43]. Generally, analysis of faeces is likely to yield a reliable picture of the diet of aerial insectivores, since earlier findings of the experimental feeding of a nestling Barn Swallow conducted by Waugh [1] showed that the proportions of different prey types ingested (including some soft-bodied prey types such as small Diptera) and the proportions recovered in the faeces are in very close agreement. This essentially means that no significant differential digestion exists between prey types with soft bodies and flexible wings and heavily chitinised prey [1]. In addition, the results of our previous field studies on insect fauna in various crop types and non-crop habitats situated in a neighbouring farming area did not indicate that any major insect group was missing from the faecal samples, which might have been expected based on these collections [11]–[16], [31]. Hence, it is worth noting that in comparison to the modern molecular techniques for avian diet sampling, which does not allow a precise assessment of the number of consumed prey items, cf. [44], the analysis of faeces can yield quantitative, taxonomic or trophic characteristics of the prey consumed.

Initially, we identified invertebrate prey items to the lowest possible taxonomic level in all individual faecal sacs. The highly aggregated, taxonomically diverse invertebrate community in an agricultural landscape, e.g. [17], [45], [46] is severely reduced during harvest, reviewed in [24]. In line with the aim of our study, to assess the seasonal differences in the dietary composition of the three target species, including the period(s) of crop harvests, we assumed that the taxonomic composition of the prey alone (i.e. the categorization of prey taxa to the level of order/genera) would be too fragmented and of no use for understanding fully the changes to the structure of the prey community. Thus, in order to provide an adequate description of these changes and a meaningful biological interpretation, we assigned the identified individual prey species/taxa to functional invertebrate groups, taking into account their relationship with the landscape and agricultural activities as the habitat of their development and their association with crop or non-crop habitats, and finally the ecological services provided to agriculture by the prey species. The classification of prey taxa was based on extensive ecological studies on invertebrate groups, including flying and ground-dwelling insects, conducted in the study area since 1960 [11]–[16], [47], see the review in [48], as well as more general knowledge in the case of pest invertebrates [49]. In addition, we took into consideration our results on the dietary composition of the three insectivores from this area from the previous year, especially the large contribution of two species of oil-seed rape insect pests [31]. Previous studies of Barn Swallows underlined the importance of the manure from large farm animals, which provide a specific community of dung-inhabiting insect prey with a relatively large body size [29], [40], [42], [50]. In view of all of the above, we assigned all the identified prey taxa into six functional invertebrate groups: 1) oil-seed rape pests; 2) pests of other arable crops, i.e. feeding/developing in broad-leaved crops, cereals, vegetables and alfalfa cultivation; 3) other crop-provisioned invertebrate taxa (various invertebrates, including predatory insects living in crop habitats); 4) coprophilous/coprophagous insects (as dung/manure-feeding beetles and some large dipterans; hereafter referred to as ‘coprophilous taxa’); 5) invertebrates from non-crop habitats (various food guilds associated with woodland/permanent vegetation); 6) invertebrates from mixed crop/non-crop habitats (occurring in both these habitats). Both pest groups and other crop-provisioned invertebrates constituted a relatively consistent group of invertebrates living in crop habitats, hereafter called ‘crop-provisioned invertebrates’ when describing the general dietary composition. Furthermore, with the exception of the group of invertebrates from non-crop habitats, all the functional invertebrate groups can formally be pooled into invertebrate resources that are agriculturally subsidized, i.e. exhibit some dependence on agricultural activities. A detailed classification of all identified prey taxa is presented in S2 File.

The dietary composition of individual faecal sacs was expressed as the number, biomass (both used in the subsequent statistical analyses) and percentage composition of the six functional invertebrate groups. Prey mass was expressed as the value calculated for dry mass (mg d.w.); these values were obtained from detailed measurements of insect mass based on the analysis of 479,087 individuals of different taxa of insects [13] and which were used in dietary studies of the aerial insectivores [31], [42], [43].

Our primary objective was to determine the seasonal changes in the number and biomass of the six functional invertebrate groups in the diets of Common Swift, Barn Swallow and House Martin nestlings. For a general description of the diets we used prey identified in all faecal sacs, including some items that were broken or stuck together. To assess the effects of phenological periods, including the harvest of the oil-seed rape crops (the habitat that is the source of a relatively large proportion of prey invertebrates; cf. [31] and this study), all faecal samples were grouped into various periods, principally successive half-monthly periods, but with one or two exceptions, namely, 25–30 May (when only faecal samples from House Martins were sampled), and between 15 and 20 July, during the oil-seed rape crop harvest. Overall, we have nine periods over the whole time span of faecal sac collection. However, only House Martin samples were collected in each of these periods. Barn Swallows and Common Swifts were sampled in seven and five periods respectively (sample sizes of the faecal sacs analysed are given in Fig. 1). Multivariate analysis of variance (MANOVA) was applied to compare seasonal changes ( = periods as independent variables) in the number and biomass of six functional invertebrate groups (dependent variables) identified in the individual faecal sacs. Prior to MANOVA some variables were log-transformed to meet the assumptions of normality. MANOVA was performed only for consecutive species-specific faecal sac sampling periods. To assess the seasonal differences for one individual invertebrate prey group we used post-hoc contrasts, applying Tukey’s test with the Spjøtvoll-Stoline modification for unequal sample sizes [51]. MANOVA was also applied to assess the general differences in dietary composition among the three target bird species in analogous time periods (five periods: from the second half of July to the first half of August). The statistical analyses were done using Statistica 7.0 [52] and Excel software. The probability of *P*<0.05 was assumed to be statistically significant.

## Results

**Figure 2:**
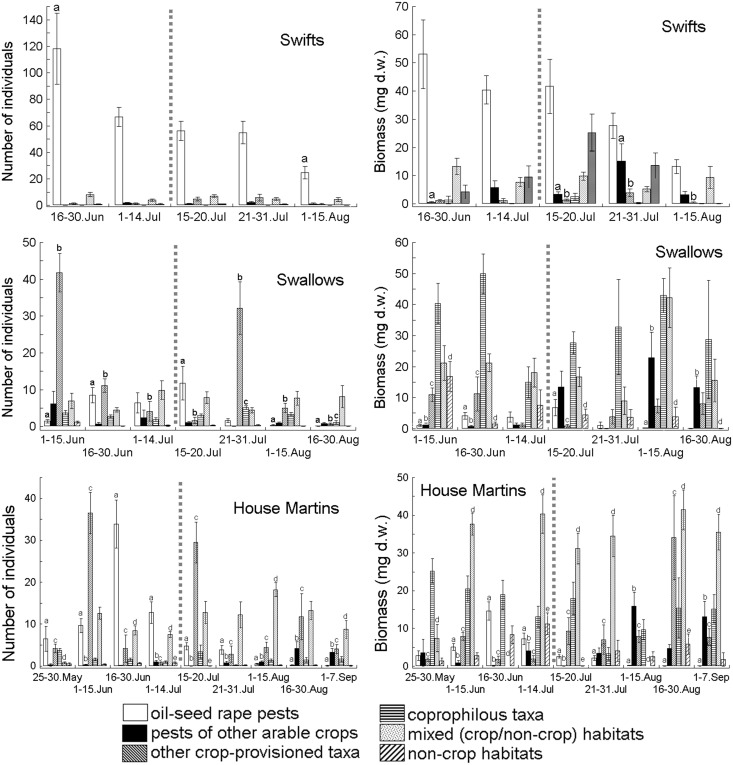
Seasonal changes in the number and biomass of six functional aerial invertebrate prey groups identified in individual faecal sacs of nestlings of Common Swifts *Apus apus*, Barn Swallows *Hirundo rustica* and House Martins *Delichon urbica* in various periods of the breeding season 2012; note different scales on y-axes; the harvest of oil-seed rape crops (15–20 July) is indicated by the vertical dotted grey line; for faecal sac sample sizes, see Fig. 1; the various letters above the bars represent significant differences between them obtained in the *post-hoc* comparison (Tukey’s test with the Spjøtvoll-Stoline modification).

Overall, in the 630 examined faecal sacs of the three bird species we identified 22,164 arthropod prey items with a total mass of 52,771 mg d.w. (Table 1; S2 File). The total from oil-seed rape pests, pests of other arable crops and other crop-provisioned taxa were the most numerous (by number) in the diets of these birds, but there were large discrepancies between the percentage distributions of their number and biomass (Table 1). Notably, a large number of other crop-provisioned taxa were small dipterans (2.6%, 56.8% and 47.6% in Swifts, Swallows and House Martins respectively) with a relatively small total biomass (S2 File). Oil-seed rape pests, including the two most numerous phytophagous taxa – *Ceutorhynchus assimilis* and *Meligethes* sp. – were the most numerous functional group of prey in Swifts (S2 File; Table 1) during the entire breeding season (Figs. 1 and 2).

**Table 1:** Total number and biomass of six functional aerial invertebrate prey groups identified in the diet of nestlings of Common Swifts *Apus apus* (*n* = 112 faecal sacs), Barn Swallows *Hirundo rustica* (*n* = 246) and House Martins *Delichon urbica* (*n* = 272) during the entire breeding season in 2012; see S2 File for a complete list of prey.

	Number of prey (%)			Biomass [mg d.w.] (%)		
Prey group	Common Swifts	Barn Swallows	House Martins	Common Swifts	Barn Swallows	House Martins
Oil-seed rape pests	5 643 *(84.1)*	1 008 *(17.8)*	2 348 *(24.0)*	3 275 *(49.2)*	502 *(2.4)*	1 137 *(4.4)*
Pests of other arable crops	119 *(1.8)*	302 *(5.3)*	298 *(3.0)*	647 *(9.7)*	1 396 *(6.8)*	1 336 *(5.2)*
Other crop-provisioned taxa	300 *(4.5)*	2 509 (44.3)	3 696 *(37.7)*	94 *(1.4)*	547 *(2.7)*	1 134 *(4.4)*
Coprophagous/-philous taxa	14 *(0.2)*	596 *(10.5)*	345 *(3.5)*	113 *(1.7)*	6 952 *(33.9)*	3 765 *(14.7)*
Mixed (crop/non-cropped) habitats	549 *(8.2)*	1 214 *(21.4)*	3 047 *(31.1)*	814 *(12.2)*	10 217 *(49.8)*	17 178 *(67.1)*
Non-cropped habitats	84 *(1.3)*	32 *(0.6)*	60 *(0.6)*	1 713 *(25.7)*	889 *(4.3)*	1 061 *(4.1)*
Totals	6 709 *(100)*	5 661 *(100)*	9 794 *(100)*	6 655 *(100)*	20 504 *(100)*	25 612 *(100)*

### Dietary effects of phenological period and crop harvest

Overall, for the set of faecal samples collected at the same time during five periods from the second half of July to the first half of August (i.e. when all three species were simultaneously present in the study area; Figs. 1 and 2) the dietary composition, expressed as the number and biomass of six functional invertebrate prey groups, varied significantly among Swifts, Swallows and House Martins (MANOVA, Wilks’s Lambda, λ_12,670_ = 0.352 and 0.225, *P*<0.0001, for number and biomass respectively).

The percentage composition of six functional aerial invertebrate prey groups in various periods of the breeding seasons were relatively more differentiated in the diets of Barn Swallows and House Martins compared to Common Swifts (Fig. 1). Visual inspection showed that the overall patterns of the percentage distribution (decrease or increase) of the six functional prey groups were quite consistent among these birds; the exception was the coprophilous insect group, which showed a relatively sharp increase in Barn Swallows after the oil-seed rape harvest (Fig. 1). Prior to the oil-seed rape harvest (15–20 July), the pests of this crop and other crop-provisioned taxa clearly decreased in all three target bird species. The changes observed in the case of the other arable pests took place in the opposite direction, their proportions in the diets of all three predator species showing a clear increase following the harvesting of the oil-seed rape. The small displacements in time of the peak of the proportion of other crop-provisioned taxa and the invertebrate group from mixed non-crop habitats between Barn Swallows and House Martins most likely resulted from the inclusion in the analysis of samples from a longer (a few days) time span (Fig. 1).

The MANOVA showed significant seasonal differences in the number and biomass of the six functional group of aerial invertebrates consumed by Swifts (Wilks’s Lambda, λ_24,217_ = 0.536 and 0.488, *P* = 0.018 and 0.0035, for number and biomass respectively), Swallows (Wilks’s Lambda, λ_36,727_ = 0.279 and 0.415, *P*<0.0001, for number and biomass respectively) and House Martins (Wilks’s Lambda, λ_48,1081_ = 0.143 and 0.216, *P*<0.0001, for number and biomass respectively) (Fig. 2).

Further post-hoc analysis of individual prey groups showed a progressive decrease in the number and biomass of consumed oil-seed rape pests in the diet of Swifts, but a significant difference was confirmed only between the two extreme periods, when the abundance of this prey group decreased nearly 5-fold (Fig. 2). The biomass of the six functional groups of prey taken by Swifts showed relatively the largest variability during the oil-seed rape crop harvest and the subsequent period (21–31 July). These changes concerned primarily other arable pests, the biomass of which increased significantly at the end of July, and decreased (5-fold in both these periods) at the beginning of August. A similar pattern was obtained for other crop-provisioned invertebrates, the biomass of which first increased 2.8-fold, then decreased 10-fold (in both cases significantly) between these three periods (Fig. 2).

Post-hoc comparisons for Barn Swallows showed a significant variation in the total number of three aerial invertebrate prey groups. One of them, the oil-seed rape pests, was relatively more abundant from the second half of June until the oil-seed rape harvest, but in August there were significantly fewer of these insects than during the oil-seed rape harvest period. Other crop-provisioned invertebrates showed two abundance peaks: in the first half of June and in late July, when the numbers of these prey items were significantly higher compared to the majority of the other periods (Fig. 2). Lastly, coprophilous insects were significantly more abundant at the end of July compared to the second half of August.

For Barn Swallows the picture of seasonal prey biomass variations was quite similar to the variation in total numbers, and four prey groups exhibited significant differences. Barn Swallows consumed a relatively larger biomass of oil-seed rape pests from the second half of June until the harvest of these plants, when their biomass was the highest, and decreased thereafter. The biomass of pests of other arable crops was highest in August and differed significantly from the first half of June. The opposite picture prevailed for the biomass of other crop-provisioned invertebrates, which was highest in June, and fell (significantly) during the oil-seed rape harvest. The biomass of invertebrates from non-crop habitats was highest in the first half of June, and clearly decreased in the subsequent periods (Fig. 2).

The abundance of four prey groups varied significantly in the diet of House Martins (Fig. 2). First, oil-seed rape pests showed a clear peak in the second half of June, and their number varied significantly from all other periods. Furthermore, oil-seed rape pests were relatively less abundant in August and September. The opposite picture was obtained for pests of other arable crops, the abundance of which was relatively low until the end of July and tended to increase markedly in August and September. Other crop-provisioned invertebrates showed two clear abundance peaks: one in the first half of June and during the oil-seed rape harvest, and another, smaller one in the second half of August. The group of prey from mixed habitats were relatively less abundant until the first period of July and in September, when their abundance was significantly smaller compared to the first half of August (Fig. 2).

The biomass of four prey groups showed significant seasonal differences in the House Martin diet. The biomass of oil-seed rape pests rose from May, peaked in the second half of June and later fell sharply; this was confirmed by the significant differences given by the post-hoc tests (Fig. 2). The biomass of pests of other arable crops was relatively the highest in August and September, its values at this time differing significantly compared to earlier periods, except for the end of May. The biomass of invertebrates from mixed habitats showed huge seasonal fluctuations, which were confirmed by the highly significant differences between neighbouring periods, such as between two periods in June or August, when values varied by as much as 41-fold. Lastly, the biomass of non-crop habitat invertebrates was significantly lower during the oil-seed rape harvest compared to the previous period and the second half of August (Fig. 2).

## Discussion

First, our study clearly demonstrates that aerial insectivores consume common crop pests of nearby crops, and appear to track crop pest availability, implying potential benefits in terms of pest reduction, reduced crop damage, and improved crop yield, but further studies aimed to evaluate these interactions are needed. Second, this study has shown that the classification into functional groups of invertebrate prey taken by the three bird species studied can be a useful tool in dietary analysis. This has yielded a biologically interpretable and relatively uniform picture of the seasonal food changes in a highly diverse taxonomic community of various prey types. Our classification has demonstrated in a practical way (it could be applicable in other studies) that the relative effect of season had a varying impact on the number and biomass of individual prey groups; nonetheless, the general direction of changes (increase/decrease) in all invertebrate groups was consistent in time for the three predator species we studied. This suggests that the use of aerial invertebrate resources by these birds generally took place in accordance with the temporal dynamics of the prey population resulting from both a natural process (phenology) and an anthropogenic one (farming). In addition, we realize that either the differences in the numbers of nests of the three aerial insectivores from which faecal samples were collected or the different number of faecal sacs sampled in consecutive periods could be some confounding factors. However, our study showed close similarities as regards changes in the contributions of the main prey groups (see the comments below), including small deviations from average values representing their percentage distribution (cf. Fig. 1). This suggests that any potential bias in the assessment of dietary composition resulting from an unequal sample size or the behaviour/feeding preferences of specific individuals is relatively small. Some implications regarding the feeding ecology and conservation of aerial insectivores have also arisen out of our study; they are discussed in the context of previous research.

As expected, the three species exhibited a fairly obvious relationship – a dietary shift after oil-seed rape crop harvest from oil-seed rape insect pests to other aerial invertebrate prey groups, especially to pests of other crops. This suggests an overall potential optimization of their foraging success associated with the incorporation into their diet of more profitable prey, which is in line with optimal foraging theory [1], [4]. In practice, it should be assumed that a harvest has two main effects on the invertebrate fauna living among the crops: one, generally known for aerial insectivores, is the flushing out of adult insects, which at harvest time are abundant and relatively easily caught, cf. [29], [30]; the other is direct mortality as a result of the mechanical destruction of populations of ground-dwelling arthropods living in the crop habitat. Importantly, our study showed some species-specific differences in the seasonal contribution of preyed-upon oil-seed rape pests, which may have practical implications for their natural control by aerial insectivores. Barn Swallows and House Martins (both species with a relatively small foraging range) consumed oil-seed rape pests only before or during the harvest of this crop, which suggests that these insect pests were taken above these crops or at a short distance from them. Swifts consumed a relatively large number of oil-seed rape insect pests in late summer (August), which suggests that these birds search for food in a relatively larger area. This results in a higher rate of predation (compared to the other two species) on immigrant populations of these insects beyond the host plant/crop vegetation. Considering that the two main species of oil-seed rape pests disperse over a relatively large distance (1–2 km) from the crop vegetation to overwintering non-crop (woodland) habitats, and the high persistence of the populations of these insects in the crop area, mortality due to mechanical destruction is probably low because the immigrant population of these insects leave their host plants before mowing begins, i.e. in May-June [53], [54]. This may indirectly confirm our findings, namely, the large proportion of these insects in the diets of the target birds.

The relatively low contribution (max. up to *c.* 5% as in House Martin) of invertebrates from non-crop habitats (stenotopic species; *sensu* [17]) in the diet of the three aerial insectivores suggests the importance of agriculturally subsidized invertebrates, including the group from mixed crop/non-crop habitats ( = ‘ecotone species’) and coprophilous taxa. In particular, the coprophilous insect group supplied a relatively large biomass of prey for Barn Swallows and House Martins (see Results), which confirms the overall significance of these insects, and indirectly too, of organic farming or fertilizers as a substratum for their development [27], [40], [42], [46], [50]. Overall, it seems that the large contribution of agriculturally subsidized prey in the three target bird species may confirm the general pattern of high productivity, including insect biomass, of agricultural systems (especially high in the case of oil-seed rape cultivation) compared to non-crop habitats. In practice this also means the spillover effect of some invertebrates, as in the case of invertebrates from mixed habitats, between these two types of land-use after the crop harvest. In particular, the spillover of insects from a crop habitat may be accelerated as a result of farming activities [17] or weather conditions [55]. Further, it should be assumed that the structural simplification of agricultural areas (i.e. the lack of physical barriers such as within compact tree stands) allows these three bird species to feed at relatively high speed over a variety of such open sites. On the other hand, the presence of some local barriers (trees, hedgerows) limits the dispersal of some flying insects, resulting in their local aggregations near such structures, to which aerial foraging birds are attracted [6]–[7], [13], [56]–[58].

### 

#### 

Three major conclusions result from this study.

1. In aerial foraging insectivorous birds breeding in temperate regions, the natural variation in food abundance is probably more important in determining clutch size and growth of nestlings than variations in dietary quality [1], [3], [59], [60]. Therefore, the diversification of agro-habitats, including specific crops (like oil-seed rape) and the presence of woody borders (where airborne insects concentrate), and agricultural practices (crop harvest, organic fertilization), all of which provide a relatively abundant but spatio-temporal variable community of invertebrate prey, should be understood as a highly compartmentalized system of valuable ecological interactions, enhancing various levels of biological diversity and the persistence of the community of aerial foragers in rural areas.
2. Like other species classically accepted as important natural avian predators, aerially foraging insectivores operate in specific conditions and feed on specific pest resources, unavailable to foliage/ground foraging birds (sensu [61], [62], [63]). Hence, our results suggest that in some crops like oil-seed rape cultivations, the potential integration of the insectivory of aerial foraging birds into pest management schemes might provide economic benefits [64], [65]. As the biological control of insect pests by aerially foraging insectivorous birds is still very poorly understood in both biological and economic terms [33], [37], further studies on the origin of the resources of airborne insects and the role of aerial insectivores as agents of the biological control of crop insect pests, including the determination of depredation rates and the cascading effects of insectivory on crop damage and yield are especially advisable.
3. We advocate the need for precise descriptions of avian diets based both on number and biomass of consumed prey and the utility of a functional invertebrate group classification (cf. the ‘functional insectivory’ concept at the level of avian species in [64]) for ecological and dietary studies of farmland birds, which can result in accurate assessments of interactions with cropping systems, food web dynamics, biocontrol services and the seasonality of the food consumed.

## Supporting Information

S1 File Supplementary geospatial data with the site of sympatric breeding of three species of aerial invertebrate feeding birds (Common Swifts, Barn Swallow and House Martins) in the village of Gołębin Stary, south-western Poland, where the dietary study in 2012 was conducted.
(DOC)

S2 File List of invertebrate prey items taken by three species of aerial feeding birds breeding in a village in south-western Poland based on the analysis of faecal sacs (number given in brackets) of nestlings of Common Swifts (*n* = 112), Barn Swallows (*n* = 241) and House Martins (*n* = 276) collected throughout the breeding season (between May 25 and September 7) in 2012. Functional prey groups: coprophagous/philous taxa (Copro); invertebrates from mixed crop/non-crop habitats (Mixed); invertebrates from non-crop habitats (Non-crop); pests of oil-seed rape (Rape); pests of other arable crops (Other pests); Other crop-provisioned invertebrates (Crop).
(DOC)
