# Supplementary material for: Functional Invertebrate Prey Groups Reflect Dietary Responses to Phenology and Farming Activity and Pest Control Services in Three Sympatric Species of Aerially Foraging Insectivorous Birds
Source: PLoS One. 2014 Dec 15;9(12):e114906. doi: 10.1371/journal.pone.0114906 (PMC4266629; doi:10.1371/journal.pone.0114906)
Supplement: S1 File — Supplementary geospatial data with the site of sympatric breeding of three species of aerial invertebrate feeding birds (Common Swifts, Barn Swallow and House Martins) in the village of Gołębin Stary, south-western Poland, where the dietary study in 2012 was conducted. (DOC) [file pone.0114906.s001.doc]

Supporting Information S1. Supplementary geospatial data with the site of sympatric breeding of three species of aerial invertebrate feeding birds (Common Swifts, Barn Swallow and House Martins) in the village of Gołębin Stary, south-western Poland, where the dietary study in 2012 was conducted:

[https://maps.google.com/maps/ms?ie=UTFandmsa=0andmsid=205043591096193698188.0004f32bba6c144db5b17anddg=feature](https://maps.google.com/maps/ms?ie=UTF&msa=0&msid=205043591096193698188.0004f32bba6c144db5b17&dg=feature)
