# Supplementary material for: Functional Invertebrate Prey Groups Reflect Dietary Responses to Phenology and Farming Activity and Pest Control Services in Three Sympatric Species of Aerially Foraging Insectivorous Birds
Source: PLoS One. 2014 Dec 15;9(12):e114906. doi: 10.1371/journal.pone.0114906 (PMC4266629; doi:10.1371/journal.pone.0114906)
Supplement: S2 File — List of invertebrate prey items taken by three species of aerial feeding birds breeding in a village in south-western Poland based on the analysis of faecal sacs (number given in brackets) of nestlings of Common Swifts (n = 112), Barn Swallows (n = 241) and House Martins (n = 276) collected throughout the breeding season (between May 25 and September 7) in 2012. Functional prey groups: coprophagous/philous taxa (Copro); invertebrates from mixed crop/non-crop habitats (Mixed); invertebrates from non-crop habitats (Non-crop); pests of oil-seed rape (Rape); pests of other arable crops (Other pests); Other crop-provisioned invertebrates (Crop). (DOC) [file pone.0114906.s002.doc]

Supporting Information S2. List of invertebrate prey items taken by three species of aerial feeding birds breeding in a village in south-western Poland based on the analysis of faecal sacs (number given in brackets) of nestlings of Common Swifts (*n* = 112), Barn Swallows (*n* = 241) and House Martins (*n* = 276) collected throughout the breeding season (between May 25 and September 7) in 2012. Functional prey groups: coprophagous/philous taxa (Copro); invertebrates from mixed crop/non-crop habitats (Mixed); invertebrates from non-crop habitats (Non-crop); pests of oil-seed rape (Rape); pests of other arable crops (Other pests); Other crop-provisioned invertebrates (Crop).

| Taxa / species | Individual mass (mg d.w.) | Functional prey group | Swifts | Swallows | House Martins |
| --- | --- | --- | --- | --- | --- |
| Araneae unident. | 2.7 | Mixed | 1 | 1 | 34 |
| Aphididae | 0.1 | Other pests | - | 205 | - |
| Thysanoptera | 0.01 | Other pests | - | - | 1 |
|  |  |  |  |  |  |
| Diptera unident. (small) | 0.2 | Crop | 160 | 2170 | 3022 |
| Syrphidae | 9.2 | Mixed | 1 | 58 | 65 |
| Calliphoridae | 14.1 | Copro | 7 | 445 | 231 |
| Tachinidae | 3.4 | Mixed | - | 70 | 53 |
| Muscidae | 5.8 | Mixed | - | 15 | 5 |
| Opomyzidae | 0.7 | Crop | - | 1 | 28 |
| Scatophagidae | 6.1 | Copro | - | 1 | - |
| Dolichopodidae | 0.9 | Mixed | - | - | 1 |
| *Chloromyia formosa* | 8.5 | Mixed | - | - | 1 |
| Brachycera | **-** | Mixed | **-** | 75 | 24 |
| **Diptera** (total) | **-** | **-** | **168** | **2835** | **3430** |
|  |  |  |  |  |  |
| *Ceutorrhynchus assimilis* | 0.7 | Rape | 3398 | 329 | 659 |
| *Ceutorrhynchus* sp. | 0.8 | Rape | - | - | 1 |
| *Otiorrhynchus* sp. | 37.3 | Other pests | 5 | - | - |
| *Apion* sp. | 0.5 | Other pests | 4 | 7 | 77 |
| *Phyllobius* sp. | 3.7 | Non-crop | - | 1 | - |
| *Curculio* sp. | 37.3 | Non-crop | - | 12 | 1 |
| *Polydrosus* sp. | 4.7 | Mixed | 4 | 20 | 161 |
| *Sitona* sp. | 4.7 | Other pests | 3 | 1 | 19 |
| Curculionidae unident. | 2.8 | Mixed | 6 | 27 | 31 |
| **Curculionidae** (total) | **-** | **-** | **3 420** | **397** | **949** |
|  |  |  |  |  |  |
| *Lema melanopa* | 3.4 | Other pests | 93 | 30 | 74 |
| *Lema* sp. | 3.1 | Other pests | 1 | - | - |
| *Chaetocnema* sp. | 0.9 | Other pests | 20 | - | 21 |
| *Psylliodes* sp. | 1.8 | Other pests | 8 | 2 | 77 |
| *Phyllotreta* sp. | 0.5 | Other pests | 8 | - | 8 |
| Chrysomelidae unident. | 7.2 | Mixed | - | **-** | 1 |
| **Chrysomelidae** (total) | **-** | **-** | **130** | **32** | **181** |
|  |  |  |  |  |  |
| *Coccinella septempunctata* | 13.7 | Mixed | - | 1 | 433 |
| *Coccinella* sp. | 7.2 | Mixed | 1 | - | - |
| *Propylaea* *14-punctata* | 3.2 | Mixed | - | 11 | 120 |
| *Adalia 10-punctata* | 3.2 | Mixed | - | - | 1 |
| *Calvia* sp. | - | Mixed | 1 | - | 6 |
| *Harmonia axyridis* | *-* | Mixed | - | - | 26 |
| *Hippodamia 13-punctata* | 5.3 | Mixed | - | - | 1 |
| *Micraspis* sp. | 1.9 | Mixed | - | - | 4 |
| *Halyzia* sp. | 3.1 | Mixed | - | - | 1 |
| **Coccinellidae** (total) | - | - | **2** | **12** | **592** |
|  |  |  |  |  |  |
| *Tachyporus* sp. | 0.5 | Crop | 63 | 39 | 15 |
| *Oxytelus* sp. | 0.3 | Crop | 1 | 139 | 67 |
| *Philonthus* sp. | 1.4 | Copro | 2 | 22 | 54 |
| *Acidota* sp. | 0.4 | Crop | - | 2 | 6 |
| *Lathrobium* sp. | 1.6 | Crop | 6 | 8 | - |
| *Heterothops* sp. | 0.3 | Crop | 2 | 17 | 2 |
| *Aleochara* sp. | 0.2 | Crop | - | 31 | 32 |
| *Stenus* sp. | 1.2 | Mixed | - | 1 | 1 |
| *Staphylinus* sp. | 32.8 | Mixed | - | 1 | 7 |
| *Haploglossa* sp. | 0.5 | Crop | - | - | 3 |
| Staphylinidae unident. | 1.8 | Mixed | - | - | 10 |
| **Staphylinidae** (total) | **-** | **-** | **74** | **260** | **197** |
|  |  |  |  |  |  |
| *Calathus* sp. | 17.0 | Mixed | - | 1 | 1 |
| *Amara* sp. | 8.5 | Crop | 1 | 36 | 52 |
| *Bembidion* sp. | 1.2 | Crop | 11 | 46 | 151 |
| *Poecilus* sp. | 26.1 | Crop | - | 1 | 7 |
| *Pterostichus* sp. | 54.2 | Crop | - | - | 2 |
| *Demetrias atricapillus* | 1.0 | Mixed | 1 | - | - |
| *Lorocera* sp. | 6.6 | Mixed | - | 4 | 52 |
| Carabidae unident. | 23.6 | Mixed | 1 | - | - |
| **Carabidae** (total) | **-** | **-** | **14** | **88** | **265** |
|  |  |  |  |  |  |
| *Cryptopleurum* sp. | 0.5 | Crop | - | 4 | 8 |
| *Cercyon* sp. | 1.1 | Copro | 4 | 39 | 5 |
| *Helophorus* sp. | 0.3 | Mixed | 10 | 25 | 29 |
| Hydrophilidae | 1.0 | Mixed | - | 10 | 2 |
| *Hister* sp. | 7.0 | Copro | - | 13 | 1 |
| Histeridae | 3.9 | Copro | - | 4 | - |
| *Silpha* sp. | 26.0 | Copro | - | - | 3 |
| *Agriotes* sp. | 9.7 | Crop | - | 7 | 3 |
| *Selatosomus* sp. | 21.0 | Crop | - | - | 1 |
| Elateridae | 13.8 | Other pests | 1 | - | 1 |
| *Meligethes* sp. | 0.4 | Rape | 2240 | 679 | 1688 |
| *Glischrochilus* sp. | 4.5 | Non-crop |  |  | 1 |
| Nitidulidae | 1.5 | Mixed | - | 1 | - |
| *Atomaria* sp. | 0.1 | Mixed | - | 2 | 10 |
| *Cryptophagus* sp. | 0.3 | Mixed | - | - | 5 |
| Cryptophagidae | 0.3 | Mixed | 2 | 1 | 13 |
| *Stilbus* sp. | 0.5 | Mixed | 3 | 3 | 4 |
| *Cartodere* sp. | 0.1 | Mixed | - | - | 2 |
| *Corticarina* sp. | 0.2 | Crop | 1 | 4 | 18 |
| *Notoxus* sp. | 1.3 | Crop | 2 | - | - |
| *Aphodius* sp. | 6.7 | Copro | 1 | 69 | - |
| *Aphodius fossor* | 6.7 | Copro | - | - | 51 |
| *Onthophagus* sp. | 9.7 | Copro | - | 3 | - |
| *Phyllopertha* sp. | 17.4 | Non-crop | - | - | 15 |
| *Psammoecus* sp. | 0.1 | Non-crop | - | 2 | - |
| *Heterocerus* sp. | 1.0 | Non-crop | - | 3 | - |
| *Rhantus* sp. | 58.7 | Non-crop | - | 1 | - |
| *Agabus* sp. | 17.0 | Non-crop | - | - | 2 |
| *Coelambus* sp. | 9.7 | Non-crop | - | - | 1 |
| Buprestidae | 5.4 | Non-crop | - | 1 | - |
| *Malachius* sp. | 2.2 | Mixed | - | - | 1 |
| *Dolichosoma lineare* | 0.6 | Mixed | - | - | 1 |
| *Cantharis* sp. | 11.2 | Mixed | - | - | 1 |
| Coleoptera unident. | 6.7 | Mixed | - | 90 | 79 |
| **Coleoptera other** (total) | **-** | **-** | **2264** | **961** | **1945** |
|  |  |  |  |  |  |
| *Thersilochus* sp. | 0.3 | Crop | 32 | 6 | 234 |
| Ichneumonidae unident. | 2.5 | Mixed | 169 | 291 | 690 |
| **Ichneumonidae** (total) | - | **-** | **201** | **297** | **924** |
|  |  |  |  |  |  |
| *Formica* sp. | 1.2 | Mixed | - | 15 | 1 |
| Formicidae | 0.6 | Mixed | 13 | - | 1 |
| *Camponotus* sp. | 1.2 | Non-crop | 13 | - | 11 |
| *Lasius* sp. | 0.6 | Mixed | 78 | 209 | 311 |
| *Myrmica* sp. | 1.2 | Mixed | 2 | 6 | - |
| Myrmicidae | 1.2 | Mixed | 2 | - | 1 |
| **Formicidae** (total) | **-** | **-** | **108** | **230** | **325** |
|  |  |  |  |  |  |
| Eulophidae | 0.1 | Mixed | 39 | 8 | 120 |
| *Habrocytus* sp. | 0.4 | Mixed | 19 | - | 19 |
| Pteromalidae | 0.2 | Mixed | 5 | 2 | 24 |
| Torymidae | 0.1 | Mixed | 2 | 2 | 13 |
| **Chalcidoidea** (total) | **-** | **-** | **65** | **12** | **176** |
|  |  |  |  |  |  |
| Apidae | 19.8 | Mixed | - | 1 | 1 |
| *Apis mellifera* | 21.4 | Mixed | - | 13 | 2 |
| *Blacus* sp. | 0.3 | Mixed | - | - | 2 |
| Braconidae | 0.3 | Mixed | 4 | 14 | 66 |
| *Chelonus* sp. | 1.1 | Mixed | 4 | 2 | 1 |
| *Chrysis* sp. | - | Non-crop | - | 1 | - |
| *Codrus* sp. | 0.2 | Mixed | - | - | 3 |
| *Monelata* sp. | 0.03 | Mixed | - | - | 2 |
| Perilampidae | 1.1 | Mixed | - | - | 1 |
| Tenthredinidae | 9.6 | Mixed | - | - | 2 |
| **Hymenoptera** (total) | - | **-** | **8** | **31** | **80** |
|  |  |  |  |  |  |
| *Aelia* sp. | 14.3 | Crop | 1 | 31 | 30 |
| *Dolycoris* sp. | 26.6 | Non-crop | 52 | 10 | 25 |
| *Eurydema* sp. | 8.2 | Other pests | - | 2 | 1 |
| *Eurygaster* sp. | 36.3 | Other pests | 1 | 22 | 10 |
| *Palomena* sp. | 42.0 | Other pests | 1 | - | - |
| Pentatomidae unident. | 26.2 | Mixed | 2 | - | 5 |
| **Pentatomidae** (total) | **-** | **-** | **57** | **65** | **71** |
|  |  |  |  |  |  |
| *Chartoscirta* sp. | 0.8 | Mixed | - | 4 | 135 |
| Corixidae | - | Non-crop | 2 | - | - |
| *Kleidocerys* sp. | 1.6 | Mixed | - | 1 | - |
| Lygaeidae | 1.3 | Mixed | 4 | 2 | 9 |
| *Lygus* sp. | 2.0 | Mixed | 3 | - | 5 |
| Miridae | 2.2 | Mixed | - | - | 1 |
| *Nabis* sp. | 2.0 | Mixed | 32 | 6 | 9 |
| *Thyreocoris* *scarabaeoides* | 2.2 | Mixed | - | 2 | 1 |
| *Neides* sp. | 1.5 | Mixed | - | - | 1 |
| Heteroptera unident. | - | Mixed | - | 4 | - |
| **Heteroptera** (total) | **-** | **-** | **41** | **19** | **161** |
|  |  |  |  |  |  |
| Auchenorrhyncha | - | Mixed | 139 | 209 | 419 |
| *Calligypona* sp. | 0.6 | Crop | - | - | 25 |
| *Philaenus* sp. | 4.5 | Non-crop | - | - | 1 |
| *Aphrophora* sp. | 6.5 | Non-crop | 15 | 1 | - |
| **Homoptera** (total) | **-** | **-** | **154** | **210** | **445** |
|  |  |  |  |  |  |
| *Chorthippus* sp. | 40.6 | Mixed | - | 1 | 5 |
| *Tetrix* sp. | 11.4 | Mixed | - | 2 | 7 |
| *Chrysopa* sp. | 2.9 | Mixed | - | - | 1 |
| *Forficula* sp. | 11.7 | Mixed | - | 1 | 1 |
| *Ectobius* sp. | 2.0 | Non-crop | 1 | - | - |
| *Coenagrion* sp. | 10.7 | Non-crop | 1 | - | 3 |
| *Pterophorus* sp. | 4.3 | Mixed | - | 1 | - |
| Microlepidoptera | - | Mixed | - | 1 | - |
| Lepidoptera | - | Mixed | - | - | 1 |
| **Other insects** (total) | **-** | **-** | **2** | **6** | **18** |
| TOTALS (ALL PREY ITEMS) | **-** | **-** | 6 709 | 5 661 | 9 794 |
